# Assessment of grain quality in terms of functional group response to elevated [CO_2_], water, and nitrogen using a meta‐analysis: Grain protein, zinc, and iron under future climate

**DOI:** 10.1002/ece3.5210

**Published:** 2019-06-22

**Authors:** Ikhlas Al‐Hadeethi, Yan Li, Abdul Kareem H. Odhafa, Hanan Al‐Hadeethi, Saman Seneweera, Shu K. Lam

**Affiliations:** ^1^ School of Agricultural, Computational and Environmental Sciences University of Southern Queensland Toowoomba Queensland Australia; ^2^ Department of Statistics, Faculty of Administration and Economics University of Wasit Kut Iraq; ^3^ Department of Soil Chemistry and Salinity, Faculty of Agriculture University of Wasit Kut Iraq; ^4^ Centre for Crop Health University of Southern Queensland Toowoomba Queensland Australia; ^5^ National Institute of Fundamental Studies Kandy Sri Lanka; ^6^ Melbourne School of Land and Environment The University of Melbourne Parkville Victoria Australia

**Keywords:** elevated CO_2 _(e[CO_2_]), iron, meta‐analysis, nitrogen, protein, water, zinc

## Abstract

The increasing [CO_2_] in the atmosphere increases crop productivity. However, grain quality of cereals and pulses are substantially decreased and consequently compromise human health. Meta‐analysis techniques were employed to investigate the effect of elevated [CO_2_] (e[CO_2_]) on protein, zinc (Zn), and iron (Fe) concentrations of major food crops (542 experimental observations from 135 studies) including wheat, rice, soybean, field peas, and corn considering different levels of water and nitrogen (N). Each crop, except soybean, had decreased protein, Zn, and Fe concentrations when grown at e[CO_2_] concentration (≥550 μmol/mol) compared to ambient [CO_2_] (a[CO_2_]) concentration (≤380 μmol/mol). Grain protein, Zn, and Fe concentrations were reduced under e[CO_2_]; however, the responses of protein, Zn, and Fe concentrations to e[CO_2_] were modified by water stress and N. There was an increase in Fe concentration in soybean under medium N and wet conditions but nonsignificant. The reductions in protein concentrations for wheat and rice were ~5%–10%, and the reductions in Zn and Fe concentrations were ~3%–12%. For soybean, there was a small and nonsignificant increase of 0.37% in its protein concentration under medium N and dry water, while Zn and Fe concentrations were reduced by ~2%–5%. The protein concentration of field peas decreased by 1.7%, and the reductions in Zn and Fe concentrations were ~4%–10%. The reductions in protein, Zn, and Fe concentrations of corn were ~5%–10%. Bias in the dataset was assessed using a regression test and rank correlation. The analysis indicated that there are medium levels of bias within published meta‐analysis studies of crops responses to free‐air [CO_2_] enrichment (FACE). However, the integration of the influence of reporting bias did not affect the significance or the direction of the [CO_2_] effects.

## 1 INTRODUCTION

Climate change factors, including high temperature and atmospheric CO_2_ concentration ([CO_2_]), are among the most pervasive environmental changes (Mueller et al., 2016). Since the industrial revolution, the increase in [CO_2_] has been documented and is predicted to increase more in the middle of the century (IPCC, 2014). Changes in these environmental variables directly or indirectly affect plant growth, development, grain yield, and quality (Fernando et al., 2012; Panozzo et al., 2014; Thilakarathne et al., 2013). Stimulation of photosynthesis together with plant nutrient metabolism alters the grain nutrient quality of many cereals and pulses. Quantitative reviews of different studies demonstrated that elevated [CO_2_] (e[CO_2_]) stimulated the grain yields of many crops. For example, the yields of C_3_ legumes and C_4_ plants were increased by 11%–31% and 14%–54%, respectively, under e[CO_2_] (Kimball, 1983; Tubiello et al., 2007), but e[CO_2_] reduced the grain N or protein concentrations of C_3_ nonlegumes (10%–15%) and had little effect on protein concentrations of legumes (–1.4%) (Jablonski, Wang, & Curtis, 2002; Taub, Miller, & Allen, 2008). Such changes in grain N, Zn, and Fe concentrations affected nutrient requirements of all cropping systems. Furthermore, the demand for these nutrients can be modified by genetic and environmental factor cropping systems. Thus, understanding grain quality trait responses to e[CO_2_] under a range of climate stressors is required to develop adaptation strategies to inevitable climate change.

The effect of e[CO_2_] on different plant physiological processes, such as photosynthesis and stomatal conductance, is well researched (Leakey et al., 2009; Thilakarathne et al., 2013). It has been well established that elevated [CO_2_] increases photosynthetic rates (Drake, Gonzàlez‐Meler, & Long, 1997; Ehleringer & Cerling, 2002; Rosenthal & Tomeo, 2013; Yamori, Hikosaka, & Way, 2014), while stomatal conductance decreases across a range of plant species (Ainsworth & Long, 2005; Ainsworth & Rogers, 2007; Farquhar & Sharkey, 1982; Medlyn et al., 2001). Correspondingly, a number of researchers have considered the concept of food security in regard to e[CO_2_] (Ziska et al., 2012). Furthermore, an ample number of studies have documented the issue of water use efficiency under e[CO_2_] levels as well (Chun, Wang, Timlin, Fleisher, & Reddy, 2011; Keenan et al., 2013). However, the effect of e[CO_2_] on plant quality, including nutrition, has yet to be fully investigated. Through photosynthesis, plants convert CO_2_ into sugar and other carbohydrates to take up minerals and other nutrients from the soil (Loladze, 2014). Each nutrient response to e[CO_2_] largely varies between functional groups and even within the same species (Ainsworth et al., 2008). Therefore, understanding the response of each functional group to e[CO_2_] under different environmental stresses is essential to addressing global food security. Recently, Loladze (2014) demonstrated that e[CO_2_] reduced wheat grain protein and nitrogen concentrations. Similarly, studies by Taub et al. (2008), De Graaff, Van Groenigen, Six, Hungate, and van Kessel (2006), Conroy (1992), and Giri, Armstrong, and Rajashekar (2016) investigated the response of grain protein to e[CO_2_] under different N regimes. Several experiments were carried out to investigate the responses of biomass and productivity to e[CO_2_] among different functional groups (Hooper & Vitousek, 1998; Reich et al., 2004). Research shows that the effects of [CO_2_] are not just presented in cereals (Wohlfahrt, Smith, Tittmann, Honermeier, & Stoll, 2018). Wohlfahrt et al. reported an increased yield of grapevines under FACE. However, there is very limited understanding on how e[CO_2_] influences grain quality traits, such as protein, Fe, and Zn under water and nitrogen stress within a range of functional groups.

Large differences in the responses of grain yields and quality to e[CO_2_] have been reported across a number of functional groups (Kimball, Kobayashi, & Bindi, 2002). Micronutrients requirements, particularly Fe and Zn, in grain and the consequences of not having these micronutrients at the required amount are well explained by the World Health Organization. Studies have shown different impacts including child mortality, mental impairment, and anemia due to the lack of Fe and Zn in different species of food crops (Cakmak, Pfeiffer, & McClafferty, 2010). Hence, assessing the status of macronutrients in different food crops is crucial as they are documented as changing with e[CO_2_]. A number of studies have been conducted to explain lower micronutrient concentrations in cereal crops under e[CO_2_] (Erbs et al., 2010; Kimball et al., 2001; Seneweera, Blakeney, & Milham, 1996). However, there is very limited understanding of how grain protein, Zn, and Fe respond to e[CO_2_] under a range of stress conditions, particularly water and nitrogen limitations.

There have been a number of meta‐analysis studies to discuss the impact of climate change on crop quality (Baig, Medlyn, Mercado, & Zaehle, 2015; Haworth, Hoshika, & Killi, 2016; Humbert, Dwyer, Andrey, & Arlettaz, 2016; Niu et al., 2016; Sutton, 2005; Zhou et al., 2017). A number of studies have shed light on the effects of carbon dioxide [CO_2_] on agricultural crops (Buchner et al., 2015; Dietterich et al., 2015; Fitzgerald et al.., 2016). Some meta‐analyses utilized a very limited number of studies for grain quality studies (Al‐Hadeethi, Li, Seneweera, & Al‐Hadeethi, 2017). Jablonski et al. (2002) conducted a meta‐analysis to combine the data on eight reproductive traits from 159 CO_2_ enrichment studies that reported the information on 79 species. They found that crops were responsive to high [CO_2_] more than wild species. In addition, grain N was not affected by the elevated [CO_2_] concentrations in legumes but reduced significantly in most nonlegumes. Other groups of researchers performed a comprehensive meta‐analysis to explore the influence of e[CO_2_] on crop nutrients compositions (Broberg, Högy, & Pleijel, 2017; Duval, Blankinship, Dijkstra, & Hungate, 2012; Ingvordsen et al., 2016; Lam, Chen, Mosier, & Roush, 2013; Lam, Chen, Norton, Armstrong, & Mosier, 2012; Li, Niu, & Yu, 2016; Myers, Wessells, Kloog, Zanobetti, & Schwartz, 2015; Taub et al., 2008). They reported that many nutrient compositions decreased in crops under elevated [CO_2_]. Neither of those studies were concentrated exclusively on the effects of high [CO_2_] on crops nutrient composition taking into consideration of the influence of water and nitrogen fertilization. And little attention was given to the impacts of key environmental factors such as water and soil nitrogen availability on crops. The abnormal increase in nitrogen impeded the process of balancing the protein content and carbohydrate content which negatively affected the production by delaying the entry of the plant's maturation stages. Also, increasing the nitrogen of the distant boundaries of the necessary needs led grain crops to produce a crop without grain. In addition, low wetness level inhibited cell growth and led to the closure of stomatal and reduced photosynthesis, and each plant process was directly or indirectly affected by water availability. To address these issues, a meta‐analysis has been carried out to analyze the effect of e[CO_2_] on protein, zinc, and iron for five different crops under different functional groups considering different levels of water and N. The study includes five different crops: wheat, rice, maize as a cereal crops and soybean and field peas as legumes. These crops define different functional groups including cereal and legumes, along with C_3_ and C_4_ photosynthetic groups. The functional group cereals and legumes best define the issues relating to protein and micronutrients. Cereals are grown for their grains which are high in protein and carbohydrates and legumes are among the most versatile and nutritious foods available. In a recent meta‐analysis, Al‐Hadeethi et al. (2017) found that the protein concentrations in wheat diminished slightly under e[CO_2_]; however, grain yields increased. In this previous study, we examined protein concentration and grain yield in a wheat crop under three environmental factors in Australia. The analysis showed that there were decreases in the Zn concentrations of some major food crops, including staple foods, such as rice, wheat, and corn. The WHO (2017) estimated the risk of an inadequate Zn uptake for approximately 17.3% of the population worldwide, including an annual death of 433,000 children under the age of five due to Zn deficiency. Therefore, deficiencies in micronutrients are not only limited to production or biomass but also more pronounced in terms of the diets and well‐being of humans.

There are not many published studies on how [CO_2_], water, and N affect grain protein, zinc, and iron concentrations. In addition, most related studies have not been reported. There is a large knowledge gap on how crops response to [CO_2_], water, and nitrogen. In this paper, we hypothesized that grain protein, Zn, and Fe concentrations are reduced under e[CO_2_], but their responses are modified by factors, such as water stress and nitrogen availability.

## 2 MATERIALS AND METHODS

### 2.1 Data selection

In 2017, a database of the effect of [CO_2_], temperature, and nitrogen on grain protein and grain yield was created (Al‐Hadeethi et al., 2017). This database was obtained from the website of the journal scientific data (http://www.nature.com/articles/sdata201536#data-records; Dietterich et al., 2015). The investigation was focused on grain proteins and grain yields of wheat crops in Victoria, Australia, under two different [CO_2_] levels (ambient and elevated), two levels of nitrogen (low and medium), and one level of temperature (ambient). A procedure based on the *dplyr* package in R program (Wickham, 2011) was utilized to re‐arrange data from individual studies, separately, under the conditions considered in this study to make them suitable for meta‐analysis. A dataset template containing the name of study, level of [CO_2_]_,_ level of temperature, level of nitrogen, name of crop, year, city, state, country, cultivar, sowing time, and replicate was created. Limitations faced in previous studies included (a) data compiled from one place and for one crop, (b) crops being cultivated under the same field conditions, and (c) crops grown at e[CO_2_] in studies using the single [CO_2_] enrichment technology free‐air [CO_2_] enrichment (FACE). In this study, those limitations were overcome by considering several crops including wheat, rice, soybean, corn, and field peas grown in different countries such as Australia, Japan, United States, and Germany. Furthermore, the effect of diverse environmental variables (nitrogen supply and water supply) on the magnitude of the [CO_2_] effect was investigated. In addition, the effect of [CO_2_] with the aforementioned environmental factors on the concentration of the basic types of micronutrient such as protein, Zn, and Fe was examined.

The data obtained from the website of the journal scientific data were expanded. In addition, a compilation of additional data from literature using a comprehensive keyword search in various databases (Web of Science, Scopus, and Natural Resources Index) and an examination of lists of references were conducted (although there was paucity of studies that contained the effect of [CO_2_] on protein, Zn, and Fe considering different levels of nitrogen and water) with the search terms are listed in Appendix S2. This study focused on investigating grain protein, Zn, and Fe for wheat, rice, soybean, corn, and field peas in Australia, Japan, United States, and Germany under two different levels of [CO_2_] (ambient and elevated), three levels of nitrogen (low, medium, and high), and two levels of water (wet and dry). The areas were chosen because we had the full access of the relevant information data, and we were able to employ meta‐analysis to investigate those published studies. An extensive reprocessing of data to the data compatible for meta‐analysis was carried out. Conducting a meta‐analysis demands a set of clear and proportionate information about the individual studies. The following criteria were important to selecting appropriate studies to be included in this analysis. First, sample size, mean, and standard deviation or standard error had to be reported for the treatments of e[CO_2_] and a[CO_2_]. Second, crop species and experimental design were identified. Finally, for studies that did not report grain protein concentration, protein values were calculated based on a measurement of nitrogen and a conversion to protein using Equation (1), where *k* = 5.36 (Myers et al., 2014).

(1) protein(weight%)=k×nitrogen(weight%)

The different levels of [CO_2_] treatments were classified as “elevated” (CO_2_ concentration ≥ 550 μmol/mol) and as “ambient” (CO_2_ concentration ≤ 380μmol/mol). The water status was classified as “wet” (water amount include precipitation + irrigation) or as “dry” (water amount include only precipitation or without precipitation + irrigation). Nitrogen concentrations (the amount of nitrogen) were classified as “low” (nitrogen concentration equivalent to zero kg N per ha), “medium” (50 kg N/ha ≤ nitrogen concentration < 120 kg N/ha), and “high” (nitrogen concentration ≥ 120 kg N/ha). The database contained 542 observations from 135 studies, including 280 observations for wheat, 118 for rice, 40 for field peas, 88 for soybean, and 16 for corn. The database of the meta‐analysis is presented in Table S1, and it will made available online.

### 2.2 Meta‐analysis

The meta‐analysis was carried out as described by Curtis and Wang (1998) and Ainsworth et al., (2002). The response ratio representing the ratio of several measures of outcomes in the treatment group to that of the control group were estimated (Rosenberg, Adams, & Gurevitch, 2000). This analysis has the merit of estimating the effect as a proportionate alteration resulting from experimental manipulation. For summarizing the influences of [CO_2_] on ecosystems, the natural log of the response ratio has been widely used (Ainsworth et al., 2002; Curtis & Wang, 1998; Hedges, Gurevitch, & Curtis, 1999). Therefore, the natural log of the response ratio (*r* = response to e[CO_2_]/ response to a[CO_2_]) was used as a metric for the analysis. The results were reported as the percentage change under e[CO_2_] ((*r* – 1) × 100). Negative values indicated a decrease in the variable compared with the ambient status, and positive percentage changes indicate an increase in the account of e[CO_2_] conditions. In previous meta‐analyses on [CO_2_] effects, effect sizes were weighted using the inverse of pooled variance (Ainsworth & Long, 2005; Duval et al., 2012), replication (Adams, Gurevitch, & Rosenberg, 1997; Blankinship, Niklaus, & Hungate, 2011), or unweighted effect sizes (Wang, 2007). In the database of this study, the collected studies did not constantly include published variance. Furthermore, the variance‐based weighting function might result in excessive weights for some studies while weighting using replication could produce less excessive weights (Van Groenigen, Osenberg, & Hungate, 2011). Thus, the studies were weighted by replication using a function of sample size given by Equation (2).

(2) $$\text{weight}=\left(n_{a}\times n_{e}\right)/\left(n_{a}+n_{e}\right),$$

where *n*_a_ and *n*_e_ represent the number of replicates of the ambient and elevated [CO_2_], respectively (Adams et al., 1997; Van Groenigen et al., 2011; Hedges & Olkin, 1985). To calculate mean effect sizes and 95% confidence intervals, bootstrapping techniques were used. For the bootstrapping using statistical software MetaWin 2.1 (Rosenberg et al., 2000), 4,999 iterations were used. Technically, a mixed‐effects model or a fixed‐effects model is not viable for non‐parametric meta‐analytic methods based on weighting by replication. However, a fixed‐effects model had to be adopted to implement a valid bootstrapping using MetaWin. The fixed‐effect model is given by Equation (3) (Borenstein, Hedges, Higgins, & Rothstein, 2009).

(3) $$T_{i}=\mu +u_{i}$$

where *T_i_* is an observed effect in the study of *i*, *μ* is the common effect, and *u_i_* is *u_i_* is the within‐study error.

The weight assigned to each study is defined as:

(4) $$w_{i}=\frac{1}{v_{i}}$$

where $v_{i}$ is within‐study variance for study *i*.

Then, the weighted mean $\bar{T}.$ can be computed as

(5) $$\bar{T}.=\frac{\sum_{i=1}^{k}w_{i}}{\sum_{i=1}^{k}w_{i}}.$$

The variance of the combined effect is defined as:

(6) $$V.=\frac{1}{\sum_{i=1}^{k}w_{i}}$$

The standard error of the combined effect is

(7) $$SE\left(\bar{T.}\right)=\sqrt{V.}$$

The 95% confidence interval for the combined effect is computed as

(8) $$\text{Lower}\;\text{limit}=\bar{T.}-1.96*SE\left(\bar{T.}\right),$$

(9) $$\text{Upper}\;\text{limit}=\bar{T.}+1.96*SE\left(\bar{T.}\right).$$

The *Z*‐value can be computed using

(10) $$Z=\frac{\bar{T.}}{SE(\bar{T..})}.$$

For a one‐tailed test, the *p*‐value is given by

(11) $$p=1-\varphi (\left|Z\right|).$$

For a two‐tailed test, the *p*‐value is given by

(12) $$p=2[1-\left(\varphi \left(\left|Z\right|\right)\right)]$$

where φ is the standard normal cumulative distribution function.

The e[CO_2_] effects on a response variable were considered significant if the confidence interval did not overlap with zero. The means of various categorical variables were considered significantly different if their 95% confidence intervals did not overlap.

### 2.3 Techniques to assess publication bias

Although meta‐analysis provides an accurate technique to combine the effect size from all the studies to obtain a pooled estimate of the common effect size, however, if the studies are biased of all relevant studies, then the effect size will reflect this bias (Borenstein et al., 2009). Various researches indicate that studies that report comparatively high effect sizes are more probable to be published than studies that report lower effect sizes. Also, published studies have considerable opportunity to find their path into a meta‐analysis, and it is possible the bias in the literature could be reflected in the meta‐analysis also. This case is commonly called publication bias.

The issue of publication bias affects the researchers who compose a narrative review. Though, meta‐analyses and systematic reviews be given more attention, perhaps due to these advanced techniques are more accurate than other methods to synthesizing research. An approach to examining whether a review is liable to publication bias is to utilize funnel plots.

The funnel plot is a technique for presenting the connection between effect size and study size. The funnel plot was plotted with treatment effects on the X‐axis and the measure of every study's size such as inverse of variance on the Y‐axis (Light & Pillemer, 1984). To test for and assess the possible impacts of bias, we performed a random effects meta‐analysis using the metafor package (Viechtbauer, 2010) in R statistical software. Bias in the dataset was assessed using regression (Egger, Smith, Schneider, & Minder, 1997) and rank correlation (Begg & Mazumdar, 1994).

## 3 RESULTS

### 3.1 Response of protein to e[CO_2_] under different N and water

**Figure 1:**
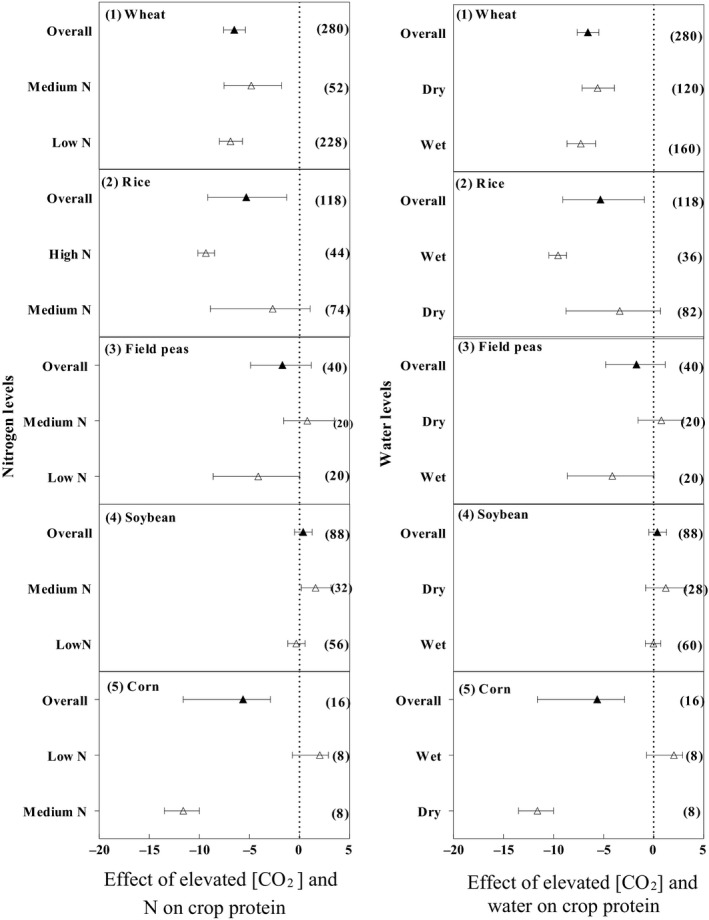
Effects of e[CO_2_] on protein for wheat, rice, field peas, soybean, and corn. Means and 95% confidence intervals are depicted. The numbers of experimental observations are in parentheses. Low N, medium N, and high N refer to nitrogen concentration equivalent to zero kg N per ha, 50 kg N/ha ≤ nitrogen concentration < 120 kg N/ha, and nitrogen concentration ≥120 kg N/ha, respectively. Wet and dry refer to the water amount including precipitation + irrigation and the water amount including only precipitation or without precipitation + irrigation, respectively

Elevated [CO_2_] significantly decreased the protein concentration in wheat (Figure 1). The average reduction in the protein concentration was 6.5% across a range of environmental conditions (Figure 1). Under low N supplies, the reduction in the grain protein concentration was 6.9% greater than the suboptimal N levels. Overall, e[CO_2_] significantly decreased the protein concentration in rice by 5.32%. Elevated [CO_2_] resulted in a small and nonsignificant reduction in protein concentration (2.69%) under medium N level, but a greater and significant reduction in protein concentration (9.36%) under high N. Overall, a small and nonsignificant reduction in the protein concentration in field peas was observed under e[CO_2_] (1.75%). The protein concentration showed a nonsignificant decrease under low N (4.12%), and there was no significant increase under medium N (0.79%). Overall, a small and nonsignificant increase in the protein concentration in soybean was observed under e[CO_2_] (0.37%). The reduction in protein concentration was nonsignificant under low N (0.33%). The increase in protein concentration was not significant under medium N (1.6%). Overall, e[CO_2_] significantly decreased the protein concentration in corn by 5.63%. The protein concentration decreased significantly under medium N (11.61%) but there was no significant reduction under low N (2.9%).

The reduction in wheat protein concentration significantly varied between the different water levels, 7.3% and 5.6% under well‐watered conditions and less well‐watered conditions, respectively. Elevated [CO_2_] resulted in a respectable reduction in protein concentration in rice by (5.31%). A nonsignificant reduction in protein concentration under dry conditions (3.38%) and a significant reduction in protein concentration under wet conditions (9.55%) were observed. Elevated [CO_2_] caused a nonsignificant decrease in the protein concentration in field peas (1.71%). The protein concentration showed a nonsignificant decrease of 4.12% under wet conditions and a nonsignificant increase under dry condition (0.79%). There was a nonsignificant increase in the protein concentration in soybean under e[CO_2_] (0.37%). The protein concentration showed a nonsignificant decrease under wet conditions (0.02%) and a nonsignificant increase under dry conditions (1.22%). Elevated [CO_2_] significantly decreased the protein concentration in corn by 5.63%. The protein concentration decreased substantially under dry condition (11.615), while a nonsignificant reduction in the protein concentration was recorded under wet conditions (2.9%).

### 3.2 Response of Zn to e[CO_2_] under different N and water

**Figure 2:**
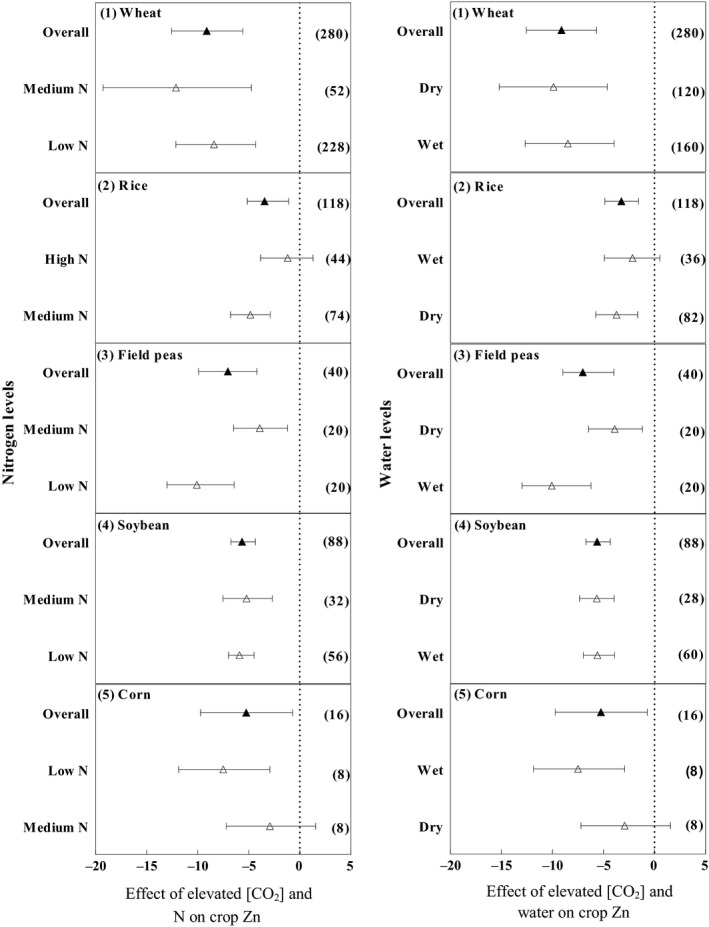
Effects of e[CO_2_] on zinc for wheat, rice, field peas, soybean, and corn. Means and 95% confidence intervals are depicted. The numbers of experimental observations are in parentheses. Low N, medium N, and high N refer to nitrogen concentration equivalent to zero kg N per ha, 50 kg N/ha ≤ nitrogen concentration < 120 kg N/ha, and nitrogen concentration ≥ 120 kg N/ha, respectively. Wet and dry refer to the water amount including precipitation + irrigation and the water amount including only precipitation or without precipitation + irrigation, respectively

Overall, the Zn concentration in wheat decreased by 9.1% under e[CO_2_] as shown in Figure 2. The reduction in the grain Zn concentration was significant at 8.4% and 12.12% for low and medium N levels, respectively. The Zn concentration in rice decreased under e[CO_2_] (3.44%). The reduction in the Zn concentration was considerable under medium N (4.82%) but nonsignificant under high N (1.18%). Elevated [CO_2_] decreased the Zn concentration in field peas (7.04%). The reduction in the Zn concentration was large under low N (10.08%) and under medium N (3.91%). Elevated [CO_2_] decreased the Zn concentration in soybean by 5.64%. The Zn concentration decreased significantly under low and medium N by 5.89% and 5.2%, respectively. Elevated [CO_2_] significantly decreased the Zn concentration in corn by 5.24%. A small and nonsignificant reduction of 2.92% in the Zn concentration under medium N was observed, but the reduction was significant under low N (7.5%).

The reduction in the wheat Zn concentration was higher under a low water level compared to high water availability. There was also a significant reduction in the Zn concentration in rice under e[CO_2_] (3.24%). Under dry conditions, the Zn concentration decreased significantly by 3.71% but was nonsignificant under wet conditions (2.15%). Elevated [CO_2_] decreased the Zn concentration in field peas significantly by 7.04%. The Zn concentration decreased significantly both under wet and dry conditions by 10.08% and 3.91%, respectively. Elevated [CO_2_] decreased the Zn concentration in soybean significantly by 5.64%. There were significant reductions in the Zn concentration under wet (5.62%) and dry conditions (5.68%). Elevated [CO_2_] significantly decreased the Zn concentration in corn by (5.24%). The Zn concentration decreased under both dry and wet conditions by 2.925% and 7.5%, respectively.

### 3.3 Response of Fe to e[CO_2_] under different N and water

**Figure 3:**
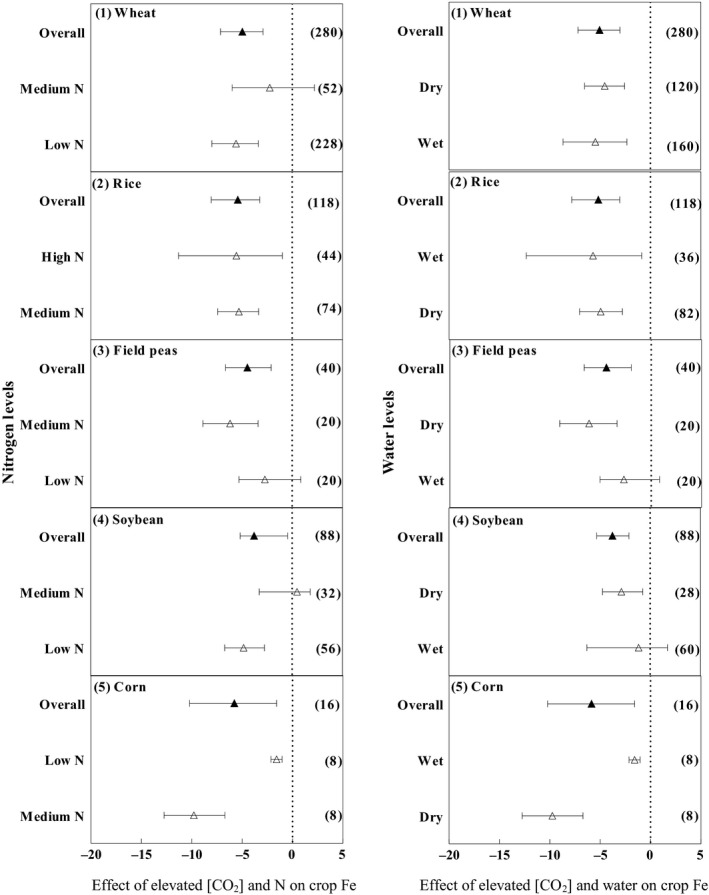
Effects of e[CO_2_] on iron for wheat, rice, field peas, soybean, and corn. Means and 95% confidence intervals are depicted. The numbers of experimental observations are in parentheses. Low N, medium N, and high N refer to nitrogen concentration equivalent to zero kg N/ha, 50 kg N/ha ≤ nitrogen concentration < 120 kg N/ha, and nitrogen concentration ≥ 120 kg N/ha, respectively. Wet and dry refer to the water amount including precipitation + irrigation and the water amount including only precipitation or without precipitation + irrigation, respectively

The Fe concentration in wheat decreased under e[CO_2_] by 4.6% (Figure 3). The reduction in grain Fe concentration was significant under low N (5.6%), but this response was not observed in medium N levels. Elevated [CO_2_] decreased the Fe concentration in rice significantly by 5.39%. Under medium and high N levels, the Fe concentration decreased significantly by 5.29% and 5.54%, respectively. Elevated [CO_2_] decreased the Fe concentration in field peas (4.44%). A small and nonsignificant reduction in the Fe concentration was observed under low N (2.7%) while a greater and significant reduction was observed under medium N (6.16%). Under e[CO_2_], the Fe concentration in soybean decreased significantly (3.77%). Additionally, the Fe concentration decreased under low N (4.81%), but there was a nonsignificant increase in the Fe concentration under medium N (1.8%). The Fe concentration in corn decreased significantly under e[CO_2_] (5.77%). Under medium and low N, the Fe concentration decreased significantly by 9.785% and 1.585%, respectively.

The Fe concentration in wheat decreases more under wet conditions (5.5%) than dry conditions (4.5%). Under e[CO_2_], the Fe concentration in rice decreased significantly by 5.17%. Reductions in the Fe concentrations under dry and wet conditions were 4.94% and 5.7%, respectively. The concentration of Fe in field peas showed a nonsignificant decrease under e[CO_2_] (4.44%). It also showed a nonsignificant decrease under wet conditions (2.7%) but a large decrease under dry conditions (6.16). The reduction in the Fe concentration in soybean under elevated [CO_2_] (2.1%) was statistically significant. The Fe concentration decreased significantly under dry conditions (3.09%), but a nonsignificant increase in the Fe concentration under wet conditions (1.1%). The reduction in the Fe concentration in corn was significant under elevated [CO_2_] (5.77%). The Fe concentration decreased substantially under dry and wet conditions by 9.78% and 1.58%, respectively.

### 3.4 Hypothetical bias

**Figure 4:**
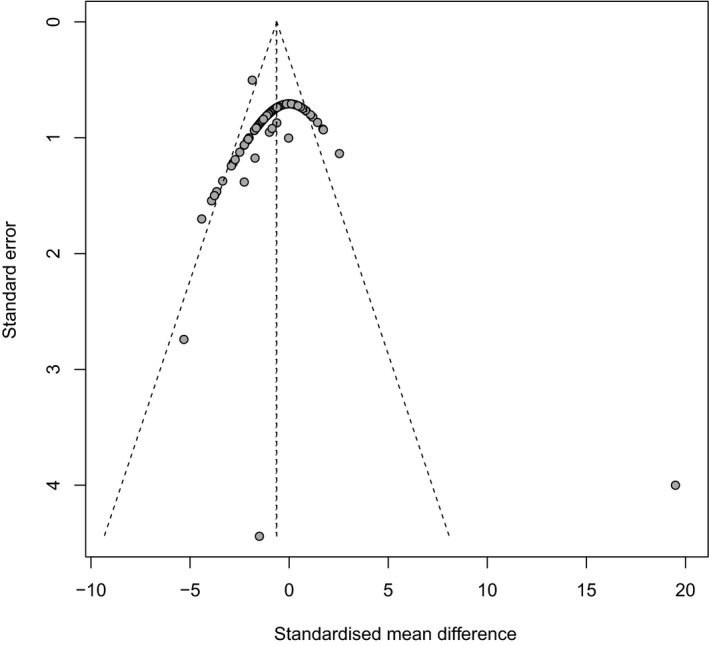
Funnel plots of crop protein (*n* = 137) show the distribution of data. Data from the studies used in the meta‐analysis are represented by solid black circles. The dashed vertical line indicates the mean effect size computed by the meta‐analysis. The funnel plot shows the Begg–Mazumdar (Begg & Mazumdar, 1994) rank correlation coefficient using Kendall's *τ* and Egger's regression test (Egger et al., 1997). Rank correlation test of asymmetry: τ = 0.552; = 0.0004; Regression test for asymmetry: z = ‐7.76; = 0.0001

**Figure 5:**
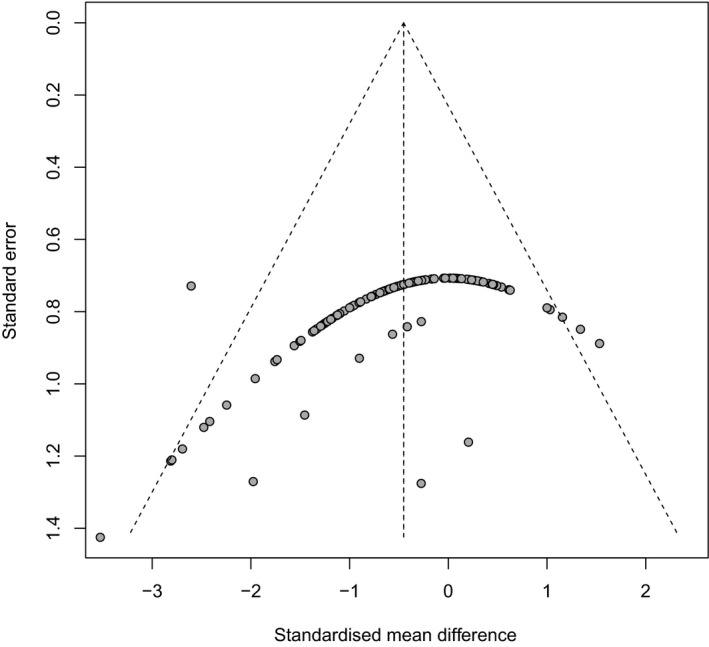
Funnel plots of crop Zn (*n* = 136) show the distribution of data. Data from the studies used in the meta‐analysis are represented by solid black circles. The dashed vertical line indicates the mean effect size computed by the meta‐analysis. The funnel plot shows the Begg–Mazumdar (Begg & Mazumdar, 1994) rank correlation coefficient using Kendall's *τ* and Egger's regression test (Egger et al., 1997). Rank correlation test of asymmetry: τ = 0; = 0.653; Regression test for asymmetry: z = ‐6.80; = 0.0001

**Figure 6:**
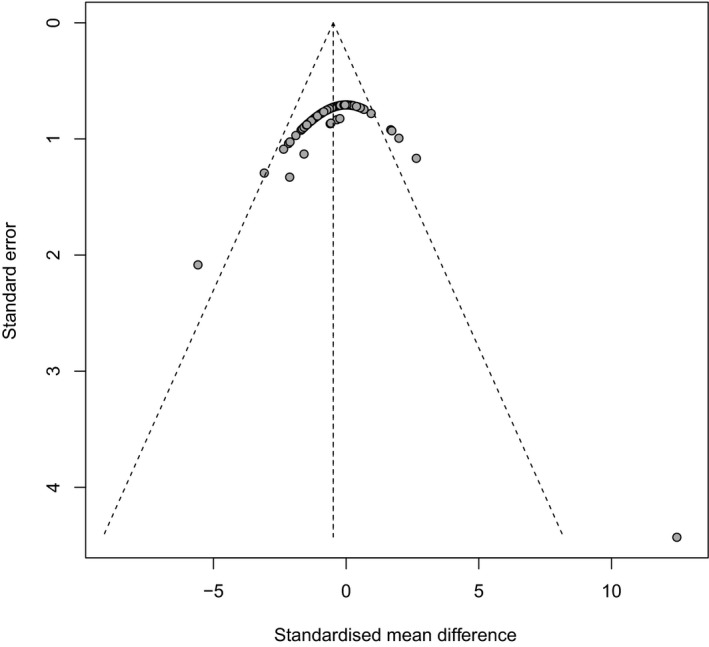
Funnel plots of crop Fe (*n* = 136) show the distribution of data. Data from the studies used in the meta‐analysis are represented by solid black circles. The dashed vertical line indicates the mean effect size computed by the meta‐analysis. The funnel plot shows the Begg–Mazumdar (Begg & Mazumdar, 1994) rank correlation coefficient using Kendall's *τ* and Egger's regression test (Egger et al., 1997). Rank correlation test of asymmetry: τ = 0; = 0.635; Regression test for asymmetry: z = ‐7.20; = 0.0001

A hypothetical publication bias induced reductions in [CO_2_] effect size of 28.02% in crop protein (Figure 4), 30.9% in crop Zn (Figure 5), and 11.23% in crop Fe (Figure 6). Our analysis is indicative of medium levels of bias within published meta‐analysis studies of crops responses to FACE. Although the integration of the influence of reporting bias did not affect the significance or the direction of the [CO_2_] effects, the outcomes of these studies should be treated with a degree of caution (Haworth et al., 2016).

## 4 DISCUSSIONS

### 4.1 Effect of CO_2_, N, and water on grain protein

The overall results were in line with our hypothesis that e[CO_2_] would reduce the protein concentration in most of the selected crops. Several studies such as Jablonski et al. (2002) and Loladze (2002) had a similar results related to a decrease in protein concentration under e[CO_2_]. The overall decreases in the protein concentrations of the selected crops were found to be more influenced by N and water content. The variations in protein concentration under low, medium, and high N levels including dry and wet water conditions showed a different response in different crops.

In most of the nonlegume C_3_ and C_4_ crops including corn, wheat, and rice, the protein concentrations decreased under medium N and dry conditions. The decreased protein concentrations in the nonlegume crops under e[CO_2_] are a consequence of decreasing protein concentrations in their photosynthetic tissues (Fangmeier, Chrost, Högy, & Krupinska, 2000; Fangmeier et al., 1999). Studies have demonstrated that a decrease in protein results from a decreased rubisco concentration (Ainsworth & Long, 2005) and a carbohydrate‐dependent decrease in the expression of photosynthetic genes (Moore, Cheng, Sims, & Seemann, 1999). In contrast to the nonlegume C_3_ and C_4_ crops, the selected legumes including field peas and soybean showed a slight increase in protein concentration under medium N and dry water conditions. The increase in nitrogen obtained in legume crops would increase protein levels. This is due to the fact that nitrogen is the main constituent of amino acids and protein acids that are the basis of proteins in the plant. In addition, water is an essential component of all these reactions and the formation of acids. Therefore, drought conditions or water shortages are the causes of a specific increase in protein concentrations. Legumes are able to use the increased carbon gained under e[CO_2_] to increase N_2_‐fixation (Allen & Boote, 2000), thus increasing grain components (Jablonski et al., 2002). Studies have shown that N_2_‐fixing legumes are typically more responsive to CO_2_ than other nonleguminous plants (Poorter, 1993; Wand, Midgley, Jones, & Curtis, 1999). Although the concentration of grain protein tends to increase slightly under low N in legumes, on average, the overall concentration of grain protein decreased. The reason for the slight increase and decrease could be that the different features of the functional group of the crops contributed to the different responses to e[CO_2_] under different N and water levels.

### 4.2 Effect of CO_2_, N, and water on grain Zn

The analysis confirmed our hypothesis related to the reduction in the Zn concentration under e[CO_2_]. Different studies have also stated that exposure to e[CO_2_] tends to reduce the concentration of mineral elements in all crops at their harvest (Fangmeier, Temmerman, Black, Persson, & Vorne, 2002). Similarly, studies have shown that CO_2_ enrichment affects nutrient uptake and distribution in a complex manner (Fangmeier, Grüters, Högy, Vermehren, & Jäger, 1997). The analysis confirms that there was a decrease in Zn concentration under e[CO_2_] in different functional group crops including legumes and nonlegume C_3_ and C_4_ crops. Furthermore, the analysis shows there was a relationship of N availability and water conditions in the reduction of the zinc concentration. The amount of N used affects the Zn concentration as smaller application of nitrogen fertilizer correlates to lower Zn grain concentrations (Cakmak et al., 2010).

### 4.3 Effect of CO_2_, N, and water on grain Fe

This study used a meta‐analysis to show the decrease in Fe concentrations for different functional groups of crops under e[CO_2_]. For Zn, the amount of N used was also found to affect the Fe concentration as a lower application of nitrogen fertilizer correlates to lower Fe grain concentrations as well (Cakmak et al., 2010).

An imbalance of different micronutrients, including Fe, is expected from e[CO_2_] as e[CO_2_] alters the leaf demand for nitrogen in different plant species (Fangmeier et al., 1997). Nitrogen fertilization makes the response of Fe in crops greater because of the presence of CO_2_. This may be due to the presence of N as a nutrient that makes the plant grow as its best. Nutrients increase the rate of the vegetative growth and increase plant activity such as photosynthesis, subsequently increasing the ability of plant to benefit from other nutrients, including Fe. This is linked to the increase in CO_2_, which is the basis of the process of photosynthesis that improves the growth and activity of the plant.

### 4.4 Assessing the publication bias

Figures 4 and 5 show that the choice of the axis representation can influence the appearance of a funnel plot. For example, the plot of crop protein and crop Fe has a clear funnel shape because there is a medium variation for the sample size. Crop Fe has a funnel shape with a little variation for the sample size as shown in Figure 6. Funnel plots should be seen as a generic means of examining whether small studies in a meta‐analysis would show larger intervention effects that may be suggestive of publication bias (Higgins and Green, 2006). However, even if small studies are associated with larger intervention effects, this may be due to other reasons rather than publication bias (Higgins and Green, 2006; Sterne et al., 2011).

## 5 CONCLUSIONS

Raising atmospheric [CO_2_] is likely to decrease protein, Zn, and Fe concentrations in many crops such as wheat, rice, and corn. However, protein and Fe concentrations increase in soybean under e[CO_2_]. Nevertheless, reduction in protein, Zn, and Fe concentrations was found to be consistent over diverse species across a wide range of experimental techniques and environmental conditions. Increased use of nitrogen fertilizers and water may lessen the effects of elevated [CO_2_] on protein, Zn, and Fe concentrations in rice. However, this approach might be only a partial solution. In other crops such as corn, high nitrogen could result in high reductions in protein, Zn, and Fe concentrations. The analysis indicated that there are medium levels of bias within published meta‐analysis studies of crop responses to FACE. However, the integration of the influence of reporting bias did not affect the significance or the direction of the [CO_2_] effects The effects of atmospheric [CO_2_] on protein, Zn, and Fe in crops are, therefore, likely to be of substantial importance to human nutrition in and beyond the 21st century. These results suggest that increased [CO_2_] under different levels of environmental conditions is likely to decrease protein, Zn, and Fe concentrations of many food crops.

## CONFLICT OF INTEREST

None declared.

## AUTHORS CONTRIBUTION

IA (64%), YL (13%), AO (8%), HA (11%). SS (2%), SL (2%). IA wrote the manuscript. YL, AO, HA, SS, and SL each contributed to the design and review of the final manuscript.

## Data Availability

The data have been deposited in Dryad. All the relevant information has been included in the data. Provisional https://doi.org/10.5061/dryad.1h1f63h.
