# Supplementary material for: Assessment of grain quality in terms of functional group response to elevated [CO2], water, and nitrogen using a meta‐analysis: Grain protein, zinc, and iron under future climate
Source: Ecol Evol. 2019 Jun 22;9(13):7425–37. doi: 10.1002/ece3.5210 (PMC6635941; doi:10.1002/ece3.5210)
Supplement: Supplementary file 2 [file ECE3-9-7425-s002.docx]

**Supporting information**

**Appendix A**

Search terms used for database collection

Google Scholar is usually used as the search platform in this study. The main combination of the search terms are:

- Increased/ elevated/ high CO_2_ , protein concentration
- Increased/ elevated/ high CO_2 ,_ zinc (Zn) concentration _March 2017 to the end of July 2017_
- Increased/ elevated/ high CO_2,_ iron (Fe) concentration
- Increased/ elevated/ high CO_2,_ grain quality and crop quality
- Increased/ elevated/ high CO_2,_ nitrogen application (N),

grain nutrient quality

- elevated/ high CO_2,_ water stress, yield quality
- elevated/ high CO_2,_ soil nitrogen and micronutrients,

nutrient composition Augest 2017 to January 2018

- elevated/ high CO_2_ , protein concentration, water stress,

nitrogen application (N)

- elevated/ high CO_2,_ grain quality and crop,

soil nitrogen and micronutrients

- Increased/ elevated/ high CO_2 ,_ zinc (Zn) concentration_,_

iron (Fe) concentration, protein concentration

- Increased/ elevated/ high CO_2,_ nutrient composition
- Increased/ elevated/ high CO_2,_ zinc (Zn) concentration_,_

iron (Fe) concentration, nitrogen application (N), January 2018 to April 2018

protein concentration

- Increased/ elevated/ high CO_2,_ zinc (Zn) concentration_,_

iron (Fe) concentration, water stress, protein concentration
